# Reference-shaping adaptive control by using gradient descent optimizers

**DOI:** 10.1371/journal.pone.0188527

**Published:** 2017-11-29

**Authors:** Baris Baykant Alagoz, Gurkan Kavuran, Abdullah Ates, Celaleddin Yeroglu

**Affiliations:** 1 Inonu University, Department of Computer Engineering, Malatya, Turkey; 2 Firat University, Department of Mechatronics Engineering, Malatya, Turkey; Lanzhou University of Technology, CHINA

## Abstract

This study presents a model reference adaptive control scheme based on reference-shaping approach. The proposed adaptive control structure includes two optimizer processes that perform gradient descent optimization. The first process is the control optimizer that generates appropriate control signal for tracking of the controlled system output to a reference model output. The second process is the adaptation optimizer that performs for estimation of a time-varying adaptation gain, and it contributes to improvement of control signal generation. Numerical update equations derived for adaptation gain and control signal perform gradient descent optimization in order to decrease the model mismatch errors. To reduce noise sensitivity of the system, a dead zone rule is applied to the adaptation process. Simulation examples show the performance of the proposed Reference-Shaping Adaptive Control (RSAC) method for several test scenarios. An experimental study demonstrates application of method for rotor control.

## Introduction

Real world control performance strongly depends on the capability of control system to adapt itself for altering dynamics of real world applications. The real control systems should deal with change of operating conditions depending on a number of factors that lead to fluctuation of system parameters. These are ageing of system components, changing environmental conditions, hardware failures, unpredictable disturbances and noise. Since the limitations of mathematical modeling techniques, mathematical models of systems cannot foresee these effects, and therefore these effects are commonly taken into consideration as parametric uncertainty, input or output disturbance model, additive white noise, etc. in the design phase of modern control systems. Through the historical development of modern control systems, numerous robust stability and disturbance rejection control woks have addressed these topics in many aspects. However, it has been understood that if a control system is not equipped with adaption skills, unpredictability and uncertainty of the real world conditions inevitably have negative impacts on the control performance of real control systems. Although static control systems, which are not skilled to adapt themselves for changing conditions, seems to work well in simulations, they cannot exhibit the same degree of control performance in real world applications. Even if a control system is designed perfectly robust for a certain degree of parametric uncertainty of plant model, it may still fail in real world conditions. Therefore, the focus of control engineering practice has been changing towards adaptive and intelligent control of systems and therefore adaptation skill and intelligence are becoming key assets of reliable and robust control of modern systems.

Adaptive control refers to the control structures that are capable of adjusting system parameters to maintain control performance in acceptable limits. When control performance is getting worse subject to changing conditions, adaptive control systems can initiate a series of adaptation operations to restore their control performance. This process is referred to as adaptation. Therefore, the control performance evaluation is a core task of the adaptation process. In general, adaptive control systems are developed to improve real world control performance of dynamic systems. In recent years, we can see some successful implications of adaptive control techniques on the control of chaotic systems. Chaotic system models allow more relevant representation of physical world, and the control of chaotic systems model is more complicated than the control of dynamic system models, which are mainly developed for requirements of industrial control systems. Progressive developments for synchronization of complex chaotic systems have been introduced by contributions of adaptive control approaches in [1–3]. Recently, a stabilization method for time-dependent strict-feedback complex variable chaotic (hyperchaotic) systems with uncertain complex parameters and perturbations has been proposed by combining Lyapunov functions of complex-valued vectors and back-stepping technique [4].

In the case of a desired control performance is described by a reference model, such control systems are often called Model Reference Adaptive Control (MRAC) systems. MRAC techniques have an analogy with supervised learning approach. A mathematical reference model as a supervisory system defines the desired input-output relation for control system, and a predetermined adaptation rule tunes adjustable parameters of the control system to resemble response of the reference model.

MRAC studies trace back to mid-1900s [5, 6]. Later, MRAC has turned into a fundamental topic of adaptive control studies [5–8]. Many works reported that MRAC approach can improve performance of control systems against unpredictable parameter variations of systems, noise and uncertain dynamics [9]. Accordingly, it has been addressed in many applications such as controlling hybrid tank systems [10], the speed estimation of the vector controlled induction motor drive [11] and controlling five-phase interior-permanent-magnet (IPM) motor drives [12], distributed control applications [13, 14], control of linkage system [15] and flight control studies [16–19]. In particular, MRAC strategy has been employed for the control applications, where system dynamics can alter according to the changing environmental condition. In literature, MRAC approach has been addressed based on many different perspectives, such as the MIT rule [8, 20], metaheuristic methods [10, 19], artificial neural network [12], Lyapunov rule [13, 20, 21], PID tuning by gradient descent optimization [22, 23], direct adaptive control techniques [24, 25], sliding mode observer-based model reference adaptive algorithm [26].

**Fig 1:**
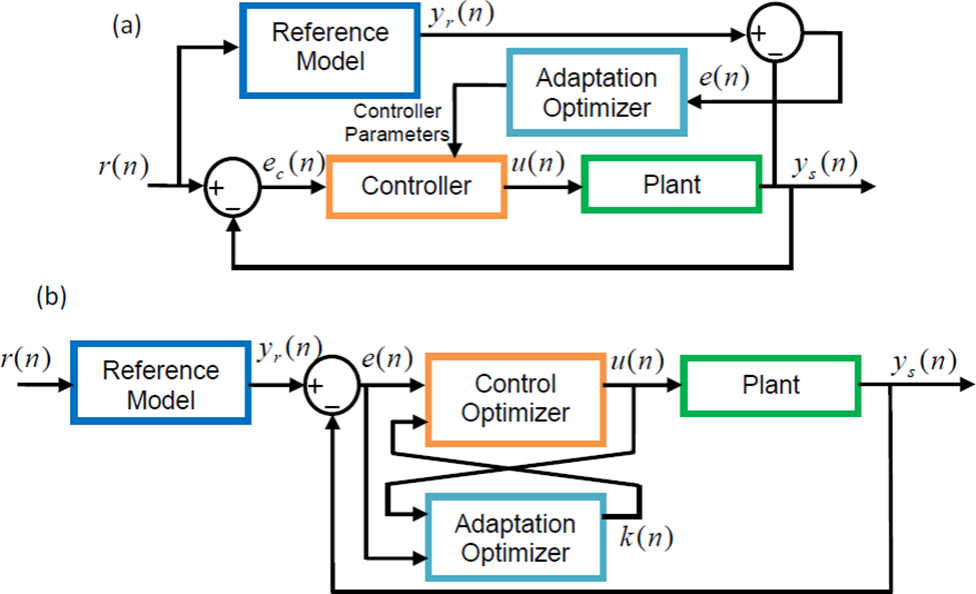
(a) Block diagrams of conventional MRAC system, (b) block diagrams of RSAC system.

In general, conventional MRAC systems contain two loops [20]: An inner loop includes an ordinary closed loop control structure consisting of a controller with tunable coefficients and a plant to be controlled. An outer loop is formed by the adjustment rule to tune adaptation parameters or controller parameters in order to reduce discrepancy between the reference model output and plant output, which is known as the model error [20]. Fig 1(A) depicts general structure of conventional MRAC structure.

In the current study, we present a single-loop approach, where both control and adaptation actions are carried out according to model error. Reference model is used to shape reference input signal and the approach is referred to as reference-shaping. Here, reference input of control system is not applied directly to the control system; instead, the reference input signal is first shaped by the reference model, then it is applied to the control system. Accordingly, the proposed adaptive controller follows only the output of the reference model and reduces the number of error loop to one that is model error. Control optimizer synthesizes control signal by using gradient descent optimization, which aims to reduce the model error in time. In addition to simulation results, which show response of the proposed adaptive control structure for various test scenarios, we conducted experimental study to demonstrate experimental performance of system.

Organization of paper is as follows: The next sections describe the system architecture of RSAC, present theoretical foundation and introduce derivations of gradient descent update rules. Afterward, numerical study is illustrated to evaluate response of system for various test scenarios. The further section presents some results obtained for rotors control experiments.

## Methodology

### System architecture and preliminaries

Fig 1 shows block diagrams of conventional MRAC system and the proposed RSAC system for comparison purpose. The main difference in structures of conventional MRAC and RSAC is that the closed loop control error, which is expressed as *e*_*c*_(*n*) = *r*(*n*)−*y*_*s*_(*n*), is not used to generate control signal in RSAC structure. Instead, *r*(*n*) signal is applied to the control loop after shaped by the reference model as in Fig 1(B). Hence, the RSAC systems perform control actions only for tracking of the reference model output. In literature, input-shaping approach (reference-shaping) was applied for vibration suppression of flexible systems, a feed-forward control based on convolution of input by a sequence of pulse was utilized [27–29].

The model mismatch between reference model and the control system is expressed by instant model error signal (*e*(*n*)) in the form of the difference between the reference system output (*y*_*r*_(*n*)) and the plant output (*y*_*s*_(*n*)).

$$e(n)=y_{r}(n)-y_{s}(n)$$ (1)

The RSAC structure includes two optimizer blocks as illustrated in Fig 1(B). One is a control optimizer to synthesize appropriate control signals for tracking of the reference model output, and the other is an adaptation optimizer that adjusts adaptation parameters according to responses of plant. Both optimizer blocks perform update rules, which work for the minimization of cost function according to gradient trends. The cost function is taken as the square of instant model error. The update equation derived for control optimizer synthesizes control signals that allow convergence of instant model error to zero. Thus, the control system output approximates to the reference model output. The update equation of adaptation optimizer works for approximation of adaptation gain to an appropriate value that characterizes instantaneous input-output relation of the plant and aids the control optimizer to generate more proper control signal so that it can follow the output of reference model.

Fully overlap of system responses of a theoretical reference model and a practical control system is not always achievable because of several practical reasons such as disparity in system dynamics, model deficiencies, uncertainties, unmodeled system dynamics and nonlinearities, structural noise, etc. In long-term, these effects cause accumulation of errors and lead to prolonging adaptation efforts that may cause the instability of adaptation equations. In order to deal with such problems, an error threshold mechanism, which permits acceptable degree of model mismatches, is defined by employing a dead-zone on the model error signal. When the amplitude of model error decreases below the error threshold, the dead-zone sets zero value to the model error. The model error is used by adaptation optimizer. In this manner, operation of adaptation process is allowed to work at the moments, when the model error exceeds acceptable limits.

Gradient descent optimization is widely used for the numerically minimization of a predefined cost function. It updates parameters according to gradient trends that allow descent of the cost function. It has popular utilization in supervised learning algorithms such as back propagation training algorithm for artificial neural network [30].

Some benefits of the proposed RSAC structure can be summarized as follows:

1. RSAC does not need a plant function presume. Hence, order of plant dynamic does not increase the complexity of update rules as seen in the following section.
2. Adaptation performance of RSAC systems strongly depends on reference tracing performance of the control loop. RSAC does not need to match time responses of reference system and control systems as in conventional two-loop MRAC structures. In some cases, due to the incompatibility of system dynamics, matching of time responses of these systems may not be possible or it may require more accurate modeling of plant dynamics. In such cases, RSAC system presents advantages because RSAC system can work in the absence of an accurate modeling of the plant and therefore update rules of RSAC are obtained more simplified and fixed than update rules of MRAC. These assets are very beneficial for control practice.

### Theoretical foundations for gradient descent update rules of reference-shaping adaptive control system

Let us express cost function as the squared difference of outputs:

$$E(n)=\frac{1}{2}e{\left(n\right)}^{2}=\frac{1}{2}{\left(y_{r}(n)-y_{s}(n)\right)}^{2}$$ (2)

An update rule for the control optimizer, which generates the control signals to minimize cost function *E*(*n*), can be written as,

$$\frac{\partial u}{\partial t}=-\eta_{c}\frac{\partial E}{\partial u},$$ (3)

where parameter *η*_*c*_ is the learning rate, and it is used to adjust the convergence speed of the gradient descent optimization method. This equation is also foundation of MIT rule. The reference model is independent of control signal, which leads to $\frac{\partial y_{r}}{\partial u}=0$. So, one can write the gradient term $\frac{\partial E}{\partial u}$ as,

$$\frac{\partial E}{\partial u}=-\frac{\partial y_{s}}{\partial u}e.$$ (4)

Let's express the instant input-output relation of a plant at the moment *t* in the form of,

$$y_{s}(t)=k(t)u(t).$$ (5)

Eq (5) states that the value of system output at any time can be written by a time-varying factor of its current input value.

*Property 1*: If the gain factor *k* is time-varying parameter, *y*_*s*_(*t*) = *k*(*t*)*u*(*t*) represents all real valued systems.

*Proof*: One always finds a value of *k*(*t*) that yields output data *y*_*s*_(*t*) ∈ *R* for an input data *u*(*t*) ∈ *R*−{0}, such that, $k(t)=\frac{y_{s}(t)}{u(t)}$. Consequently, the instant input-output relation, expressed as *y*_*s*_(*t*) = *k*(*t*)*u*(*t*), becomes valid for real valued systems.

This property reduces model dependence and hence the adaptive control method does not require a prior dynamic plant model presume. Instead, it estimates the current value of *k*(*t*) via gradient descent optimization. This can be a significant advantage for control practice. Thus, design and implementation complications originating from inaccurate modeling can be reduced.

In Eq (4), one can write $\frac{\partial y_{s}}{\partial u}=k$. Then, update rule for the control optimizer can be obtained as,

$$\frac{\partial u}{\partial t}=\eta_{c}k(t)e(t).$$ (6)

For discrete form, the finite difference is commonly used for derivative operators, and iterative update rule of control process can be written for a time sampling *t* = *nT*_*s*_ as,

$$u(n+1)=u(n)+\eta_{c}k(n)e(n).$$ (7)

In a similar manner, an update rule for the adaptation optimizer, which estimates instant adaptation gain *k*(*n*) to minimize *E*(*n*), can also be written according to the gradient descent as,

$$\frac{\partial k}{\partial t}=-\eta_{a}\frac{\partial E}{\partial k},$$ (8)

where parameter *η*_*a*_ is the learning rate for adaptation. The gradient term $\frac{\partial E}{\partial k}$ is written as,

$$\frac{\partial E}{\partial k}=-\frac{\partial y_{s}}{\partial k}e.$$ (9)

According to the Eq (5), one can write $\frac{\partial y_{s}}{\partial k}=u$ and the update rule for adaptation optimizer can be derived as,

$$\frac{\partial k}{\partial t}=\eta_{a}u(t)e(t).$$ (10)

In discrete form, an iterative scheme of update rule for adaptation process can be written as,

$$k(n+1)=k(n)+\eta_{a}u(n)e(n).$$ (11)

One of weakness of gradient descent solutions is its sensitivity to noise signal because of misleading impacts of noisy signal on gradient descent operation. The derivatives of gradient operators are sensitive to noise so that noisy spikes on signals can yield high amplitudes of derivative operators, which can results in misleading of gradient directions. On the other hand, the time response of theoretical reference model cannot exactly match the response of real systems. This is another factor that can reduce the performance of adaptation processes. In such cases, stabilization of adaptation parameters may not be possible, and lead to instability of control system as a result of overgrowing of adaptation parameters. To deal with this problem, the following dead zone rule to modify the instant model error signal is used:

$$e_{d}(n)=\{\begin{array}{cc} 0 & ,\left|e(n)\right|<e_{z}\\ e(n) & ,for\;others \end{array},$$ (12)

where *e*_*z*_ is error threshold. This equation enables to govern adaptation optimizer with respect to the level of model error. It can switch on or off adaptation process operations depending on error signal magnitude. Accordingly, the proposed system performs the adaptation only for the model errors that exceeds an unacceptable model mismatch level. This modification to model error signal can be helpful to deal with implementation problems such as the misleading impacts of inherent, low-level noise signals (quantization error, system noise, etc.) on gradient descent optimization and system dynamic mismatching problems. Considering these practical concerns, the update rule for adaptation optimizer can be enhanced as,

$$k(n+1)=\{\begin{array}{cc} k(n) & ,\left|e(n)\right|<e_{z}\\ k(n)+\eta_{a}u(n)e(n) & ,for\;others \end{array}.$$ (13)

It can be useful to discuss stability conditions of the proposed adaptive control system, theoretically. We used Lyapunov stability theorem to investigate stability conditions of the proposed RSAC method as follows:

*Remark 1*: For positive learning rates, *η*_*a*_ > 0 and *η*_*c*_ > 0, and a first order dynamic reference model in the form of $T_{R}(s)=\frac{a}{s+a}$, where *a* > 0 to ensure a stable response, output of proposed adaptive control system approximates to the output of *T*_*R*_(*s*) in time and the proposed adaptive control scheme also behaves stable.

*Proof*: To use Lyapunov stability to verify stability of system, let us take positive definite function $E=\frac{1}{2}e^{2}$ as the Lyapunov function. Here, Lyapunov theorem suggests us that if the condition of $\frac{dE}{dt}<0$ is provided, the Lyapunov function *E* converges to zero. It refers that model error decreases in time and thus output of control system approximates to the output of reference model. Obviously, the control system becomes stable as long as the first order reference model $T_{R}(s)=\frac{a}{s+a}$ is stable.

Lets us show $\frac{dE}{dt}<0$:

$$\frac{dE}{dt}=\frac{de}{dt}e=\left(\frac{dy_{r}}{dt}-\frac{dy_{s}}{dt}\right)e$$ (14)

The first order reference model, given by $T_{R}(s)=\frac{y_{r}(s)}{r(s)}=\frac{a}{s+a}$, can be expressed in the time domain as,

$$\frac{dy_{r}}{dt}=-ay_{r}+ar=a(r-y_{r}).$$ (15)

By considering Eq (5), one can write the derivative of *y*_*s*_ as,

$$\frac{dy_{s}}{dt}=\frac{d}{dt}(ku)=\frac{dk}{dt}u+\frac{du}{dt}k.$$ (16)

Then, considering the update rules $\frac{\partial u}{\partial t}=\eta_{c}ke$ and $\frac{\partial k}{\partial t}=\eta_{a}ue$, it can be written as,

$$\frac{dy_{s}}{dt}=\eta_{a}u^{2}e+\eta_{c}k^{2}e.$$ (17)

Then, by using Eqs (15) and (17) in Eq (14) one obtains,

$$\frac{dE}{dt}=\left(a(r-y_{r})-(\eta_{a}u^{2}e+\eta_{c}k^{2}e)\right)e.$$ (18)

For *a* > 0, the first-order reference model $T_{R}(s)=\frac{a}{s+a}$ is asymptotically stable, that is, for *t*→∞, the difference between reference input signal and reference model output goes to zero, (*r*−*y*_*r*_)→0, then it is evident that

$$\frac{dE}{dt}\to -e^{2}(\eta_{a}u^{2}+\eta_{c}k^{2}).$$ (19)

Since *η*_*a*_ > 0 and *η*_*c*_ > 0, the term −*e*^2^(*η*_*a*_*u*^2^ + *η*_*c*_*k*^2^) is always negative, so the stability condition $\frac{dE}{dt}<0$ is satisfied. The model error decreases in time and the control system output approximates to the output of the reference model. Here, we know that the reference model is always stable function because of $T_{R}(s)=\frac{a}{s+a}$, where *a* > 0. Consequently, the proposed adaptive control system becomes a stable system.

*Remark 2*: For strictly decreasing model error, the first-order reference model should satisfy the following condition,

$$a<\gamma (\eta_{a}u^{2}+\eta_{c}k^{2}),$$ (20)

where $\gamma =\frac{\min(y_{r}-y_{s})}{\max(r-y_{r})}$.

*Proof*: For optimality of adaptation actions, it is very useful to find sufficient conditions that make *E* a monotone and strictly decreasing function. For strictly decreasing model error in time, the condition $\frac{dE}{dt}<0$ should be satisfied for all *t* ≥ 0. By using Eq (18) for $\frac{dE}{dt}$, one can write,

$$\frac{dE}{dt}=a(r-y_{r})e-(\eta_{a}u^{2}+\eta_{c}k^{2})e^{2}<0.$$ (21)

By reorganizing this inequality, one obtains the parameter *a* that satisfies the condition $\frac{dE}{dt}<0$ as,

$$a<\frac{e(\eta_{a}u^{2}+\eta_{c}k^{2})}{\left(r-y_{r}\right)}.$$ (22)

In proper and causal control systems, output *y*_*r*_ follows reference input *r*, and output *y*_*s*_ follows output *y*_*r*_. In these cases, the sign of (*r*−*y*_*r*_) is the same as the sign of *e* = (*y*_*r*_ − *y*_*s*_). Let min(*e*) represent the minimum value of *e* and max(*r*−*y*_*r*_) represent the maximum value of (*r*−*y*_*r*_), the following inequality is valid arithmetically.

$$\frac{\min(y_{r}-y_{s})(\eta_{a}u^{2}+\eta_{c}k^{2})}{\max(r-y_{r})}\leq \frac{e(\eta_{a}u^{2}+\eta_{c}k^{2})}{\left(r-y_{r}\right)}.$$ (23)

By considering Eq (23), it is evident that parameter *a* can be chosen according to,

$$a<\frac{\min(y_{r}-y_{s})(\eta_{a}u^{2}+\eta_{c}k^{2})}{\max(r-y_{r})}=\gamma (\eta_{a}u^{2}+\eta_{c}k^{2}).$$ (24)

The Eq (24) yields a sufficient condition that makes $\frac{dE}{dt}<0$ valid for all *t* ≥ 0. Therefore, by choosing parameter *a* according to Eq (20), it can be possible to have strictly decreasing response of model error in control applications. For a practical control system, the constant $\gamma =\frac{\min(y_{r}-y_{s})}{\max(r-y_{r})}$ is almost definite and hardware limitations determine bounds of (*r*−*y*_*r*_) and (*y*_*r*_−*y*_*s*_) parameters.

## Numerical study

In this section, we performed two simulation scenarios in Matlab/Simulink environment. In the first scenario, we demonstrate effects of disturbance signal on the proposed RSAC system. In the second scenario, we investigate effects of an instant perturbation of plant function on the control performance of RSAC, and compare it with conventional MRAC.

### Simulation scenario 1

**Fig 2:**
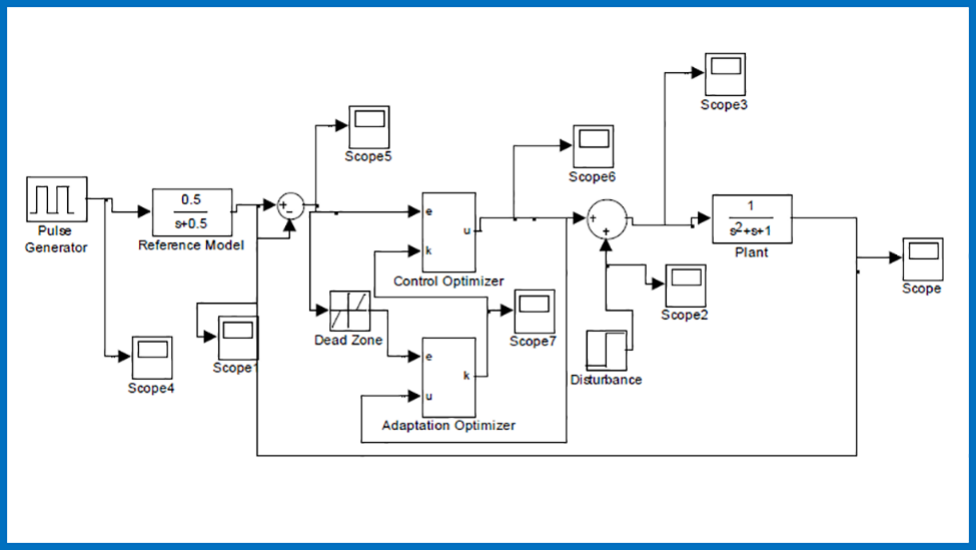
Simulink simulation model for the first scenario.

In this scenario, adaptive control of a second order plant function $G(s)=\frac{1}{s^{2}+s+1}$ according to a reference model $T_{R}(s)=\frac{0.5}{s+0.5}$ under an additive input disturbance is illustrated. It is noteworthy that orders of plant function *G*(*s*) and reference model *T*_*R*_(*s*) are different and this dynamic mismatch is a factor that complicates the adaptation process. Fig 2 shows the Simulink simulation model that was developed for this test scenario.

We applied a reference input signal with square waveform with the period of 150 sec and simulated the system for 30000 sec to confirm stability of the system via long-term observations. In simulations, the learning rates were set to *η*_*a*_ = 1.5 and *η*_*c*_ = 1. To initiate control signal generation by the recursive update rule of control process, we set a non-zero initial value for the adaptation gain *k*, that is, *k*(0) = 1. The error threshold was configured to *e*_*z*_ = 0.09 in simulations.

**Fig 3:**
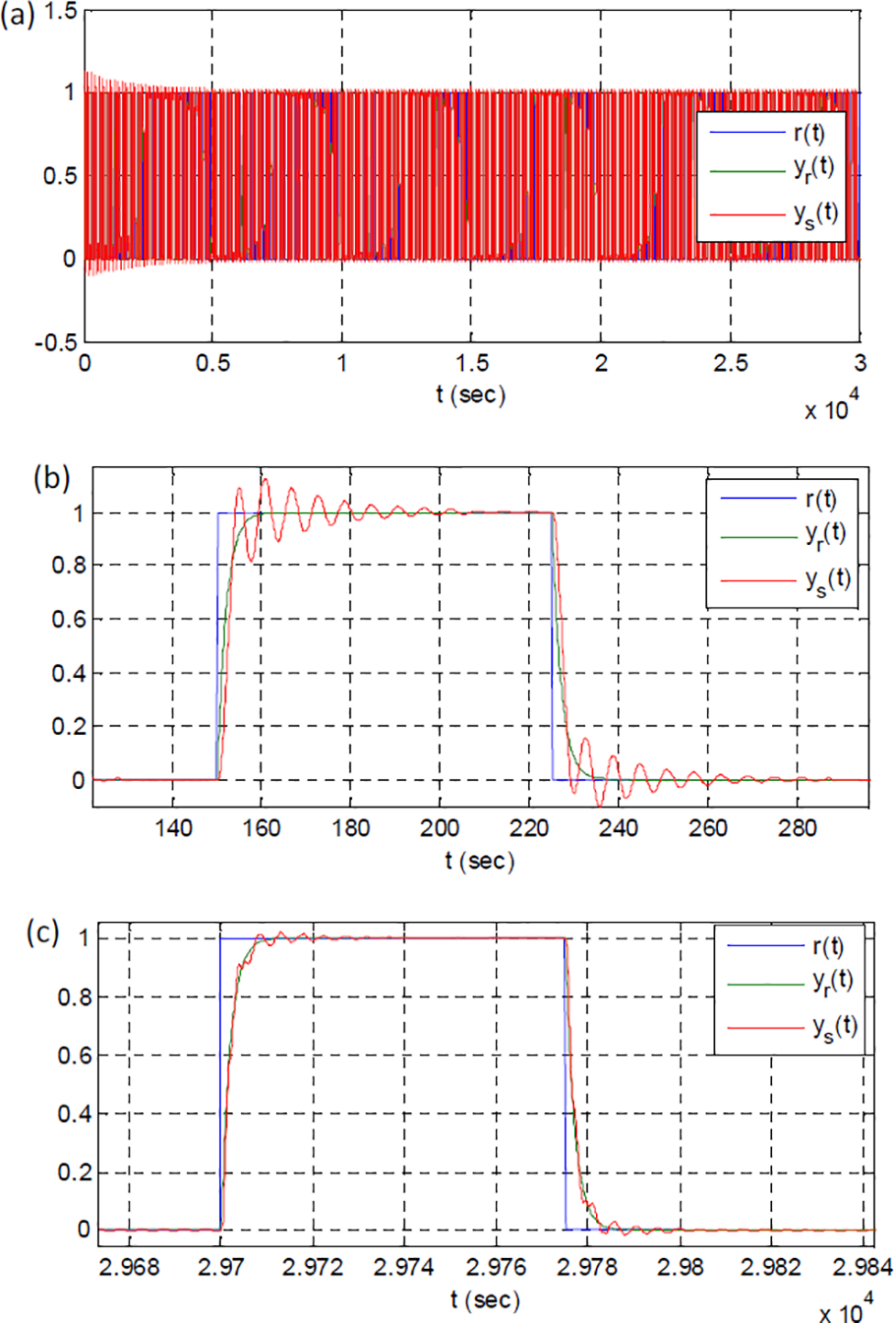
The reference input, outputs of the reference model and the plant: (a) a view of full-time simulation, (b) a close view of initial responses, (c) a close view of final responses.

Fig 3 shows the reference input, outputs of the reference model and plant. Fig 3(A) clearly validates the stability of adaptive system. Fig 3(B) and 3(C) show the initial and the final responses, respectively. These figures reveal that adaptation process takes place and the output of plant approximates to the output of reference system.

**Fig 4:**
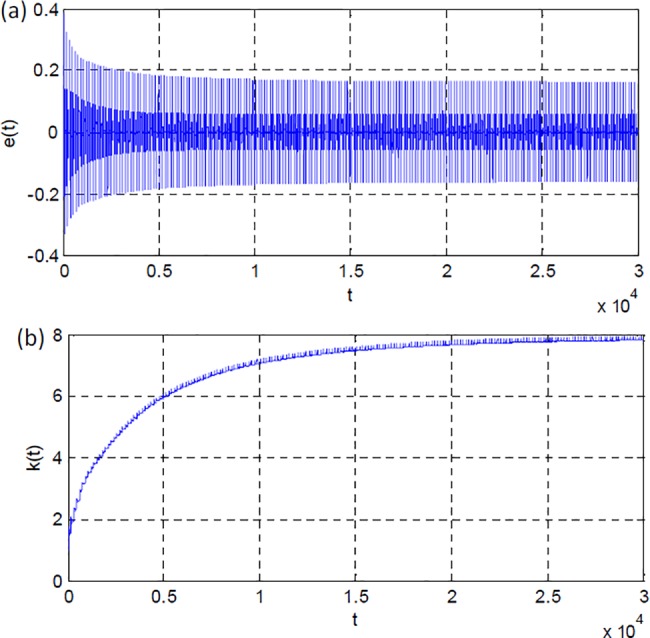
(a) Model error, (b) adaptation gain from the first simulation scenario.

Fig 4(A) and 4(B) show change of model error signal and the adaptation gain during the simulation. The adaptation gain approximates to the value of eight and stabilizes at this level.

**Fig 5:**
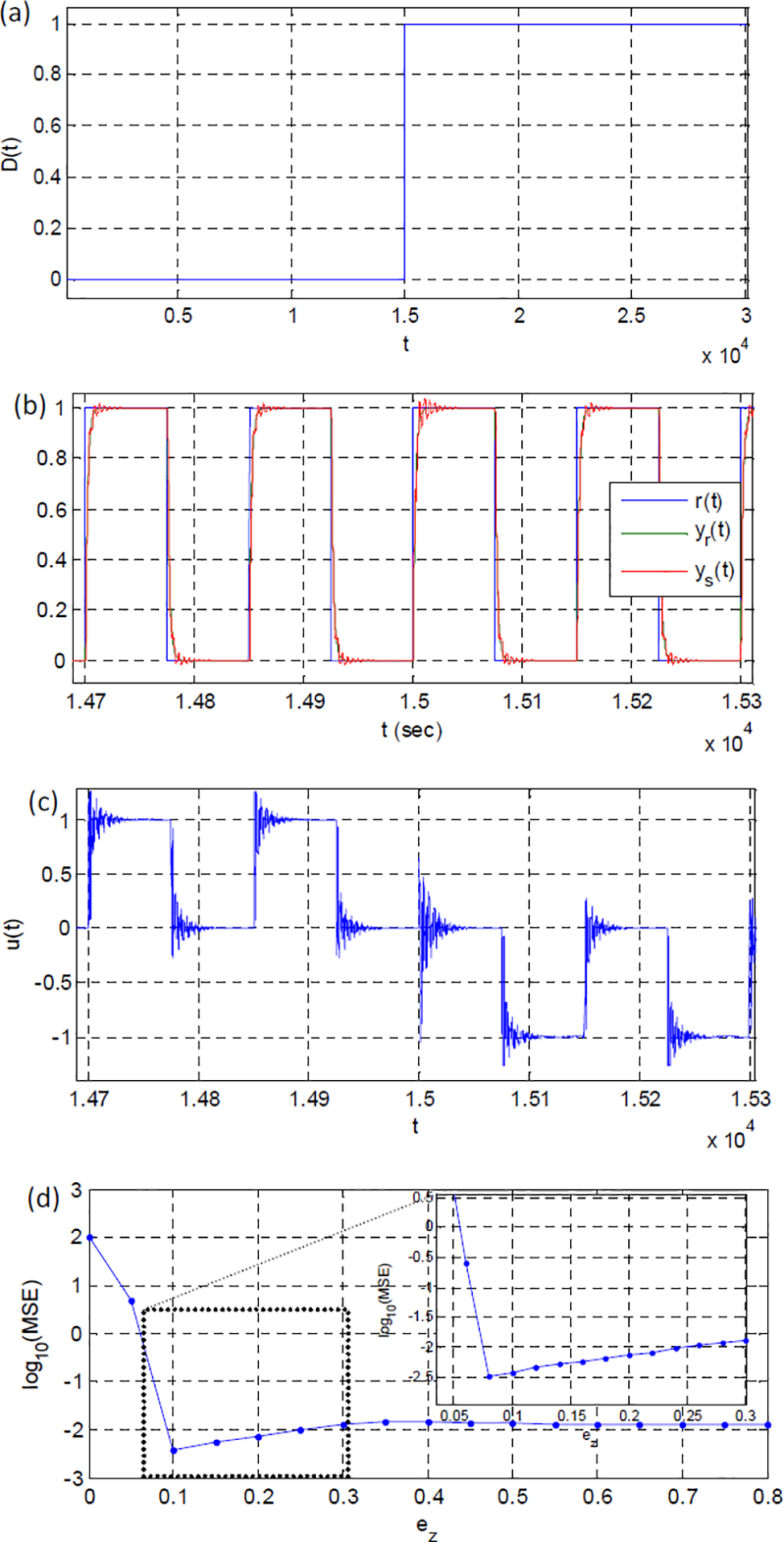
(a) Step input disturbance, (b) system outputs, (c) control signal from the first simulation scenario and (d) logarithmic scaled MSE values for various values of error threshold *e*_*z*_.

To see impacts of the step disturbance, which was applied at 15000 sec in Fig 5(A), system outputs and change of the control signal are presented in Fig 5(B) and 5(C). At the onset of step disturbance, ripples increase at plant output, however control and adaptation optimizers reduce ripples. In order to illustrate contributions of error threshold *e*_*z*_ to system performance, Fig 5(D) presents the mean square error (MSE) measured for various values of *e*_*z*_ under a band limited white noise type disturbance insertion with power of 0.01. According to the figure, an optimal value of *e*_*z*_ is 0.9 and further increasing of *e*_*z*_ leads to deterioration of adaptation performance because of unnecessarily interruptions of adaptation optimizer by larger dead zones.

### Simulation scenario 2

**Fig 6:**
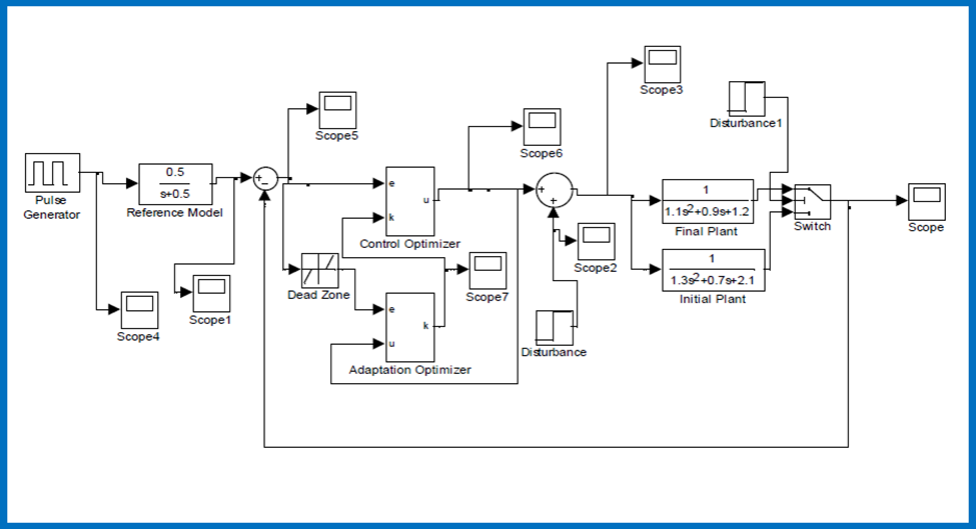
Simulink simulation model for the second scenario.

In this scenario, we investigate response of the proposed adaptive control structure for the case of an instant parametric perturbation of plant function. At the simulation time of 15000 sec, coefficients of initial plant function $G(s)=\frac{1}{1.3s^{2}+0.7s+2.1}$ is instantly altered to the plant function $G(s)=\frac{1}{1.1s^{2}+0.9s+1.2}$. During the simulation, reference model was kept as $T_{R}(s)=\frac{0.5}{s+0.5}$. The error threshold was configured to *e*_*z*_ = 0.09. The learning rates and initial value of adaptation gain were set to (*η*_*a*_ = 2, *η*_*c*_ = 1) and *k*(0) = 1, respectively. Fig 6 shows the Simulink simulation model developed for this test scenario.

**Fig 7:**
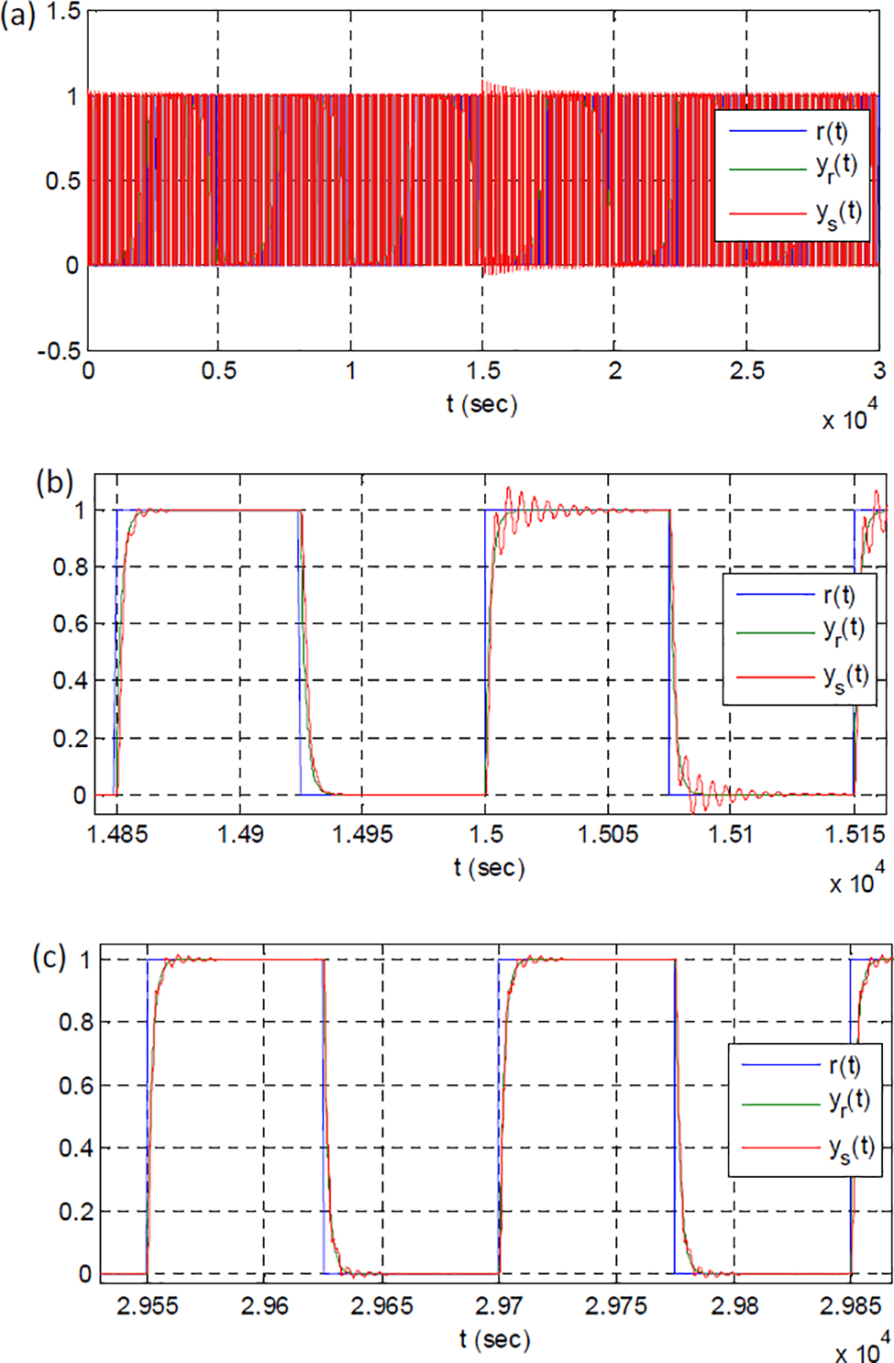
Reference input, outputs of reference model and the plant: (a) a view of full-time simulation, (b) a close view of initial response for the plant perturbation, (c) a close view of final responses.

**Fig 8:**
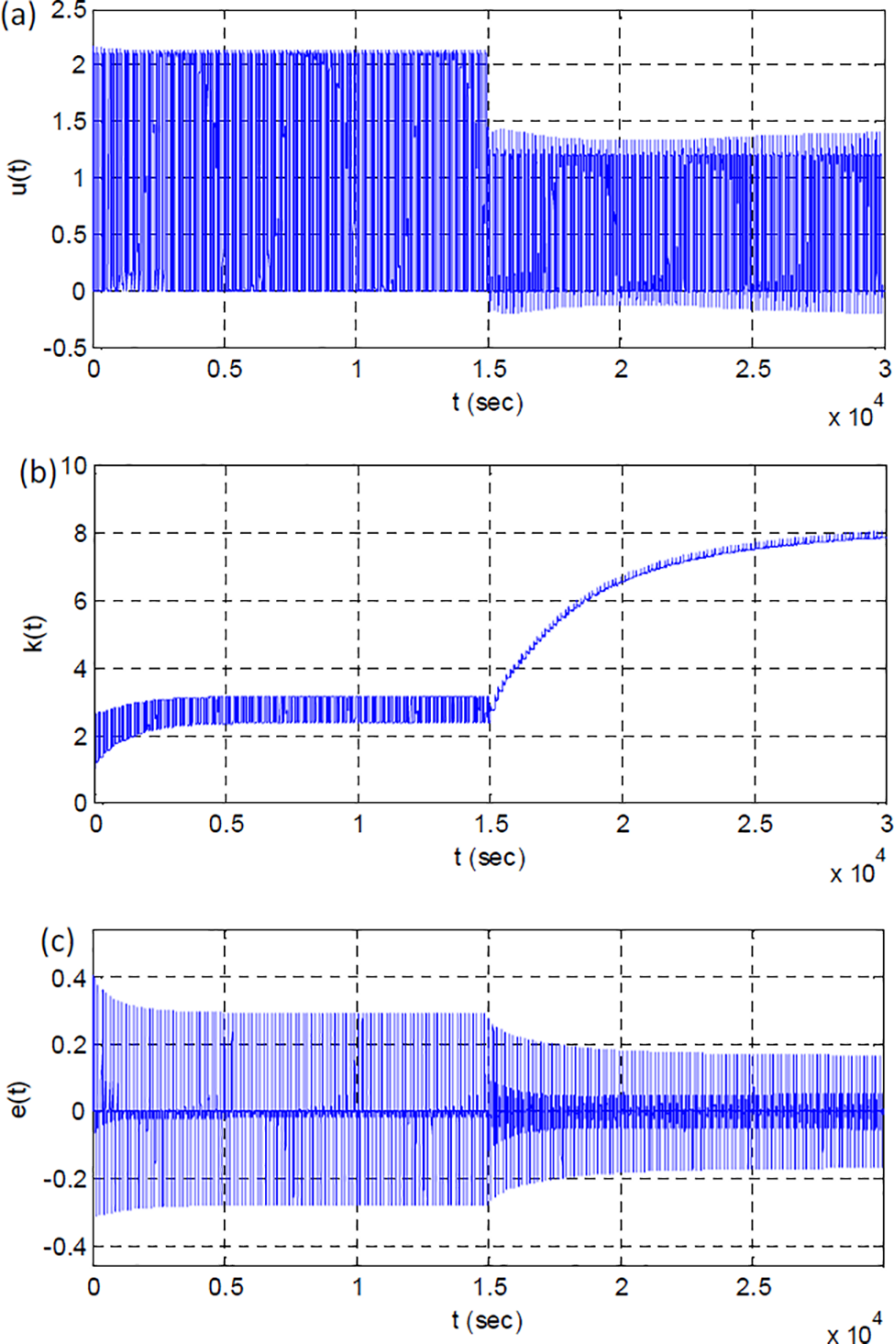
(a) Control signal, (b) adaptation gain; (c) model error signal from the second simulation scenario.

**Fig 9:**
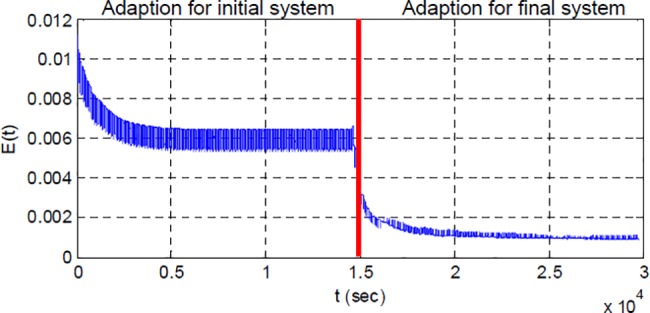
The short-time average square error calculated for the second simulation scenario.

Fig 7 shows response of RSAC system for an instant change of plant function at 15000 sec. Fig 7(A) illustrates overall response of the system during 30000 sec to observe long-term performance of system. Fig 7(B) shows impacts of plant perturbation on the output of the control system. The parametric change of plant function leads to increase of overshoot and ripples at the beginning. Fig 7(C) shows the output of plant at the end of simulation. One can see that the overshoot and ripples almost disappears. These results confirm improvement of system response by adaptation efforts, after the plant perturbation. Fig 8 shows the corresponding changes in control signal, adaptation gain and model error during the adaptation process. At the first 15000 sec, system adapted itself for the initial plant function. After the plant perturbation took place, the range and amplitude of control signal were adjusted for new plant function as shown in Fig 8(A). Change in average power of controller is an indication of alterations in system properties [31]. Power estimation of controller signal decreases from 145.7 to 51.0 at 15000 sec, and this validates system perturbation. Fig 8(B) shows change of adaptation gain that enables better fitting to reference model output. Fig 9 shows the short-time average squared model error calculated by.

$$E(t)=\frac{1}{L}\int_{t}^{t+L}e^{2}dt$$ (25)

The figure clearly demonstrates convergence of average square error for *L* = 1000 and it confirms adaptation of RSAC system for the initial plant and final plant functions in time.

**Fig 10:**
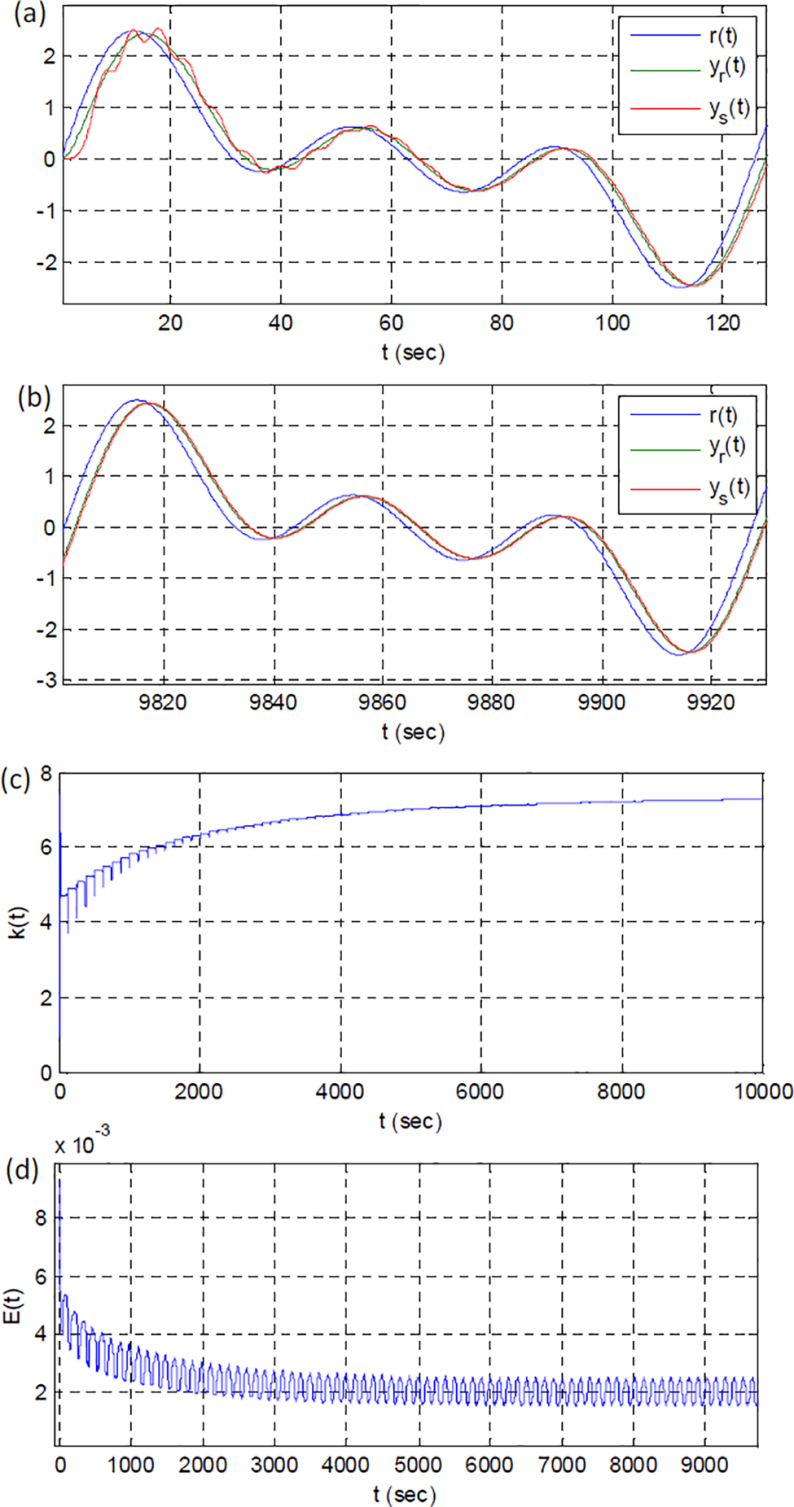
System outputs for continuous multi-sinusoidal reference input (a) at the beginning and (b) at the end of simulation, (c) adaptation gain, (d) short-time average square error.

It is useful to show performance of the adaptive control system for continuous multi-sinusoidal reference input signal. The plant function for this simulation is $G(s)=\frac{1}{2.3s^{2}+1.7s+2.3}$. Fig 10 reveals successful response of RSAC system for a continuous input signal.

**Fig 11:**
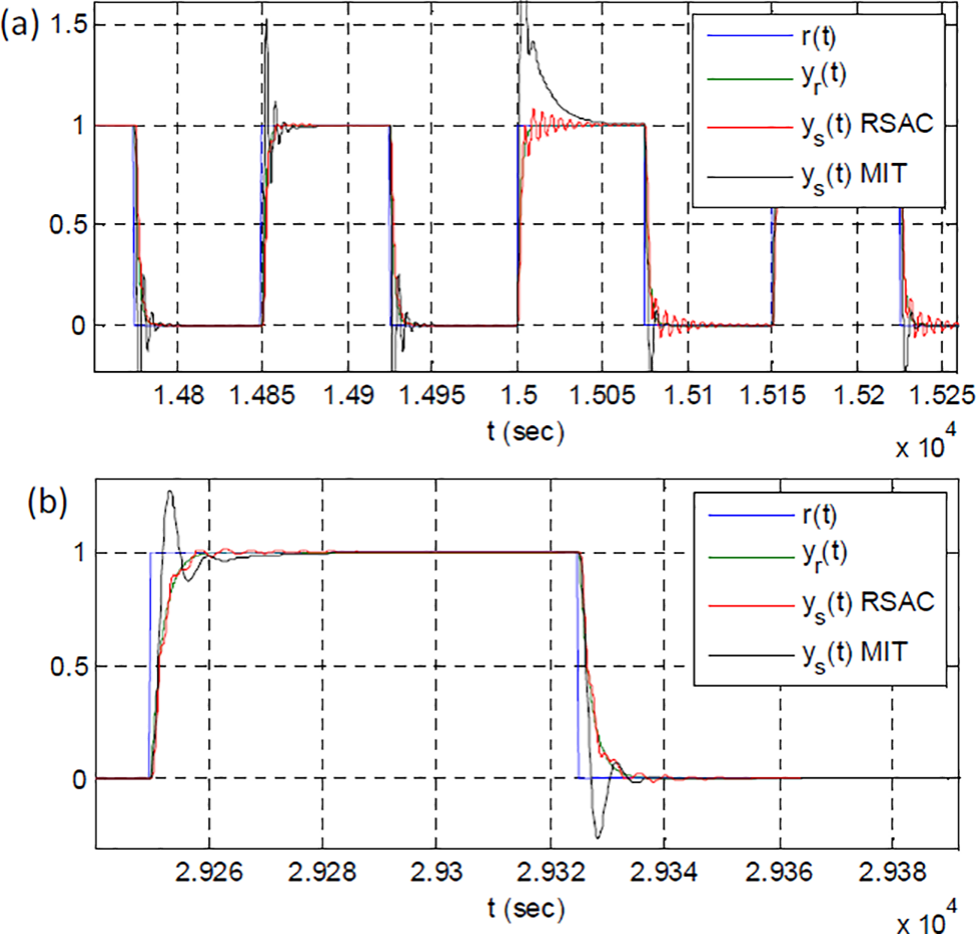
A comparison of outputs of RSAC and conventional MRAC with MIT rule: (a) a close view of responses of systems for the plant perturbation at 15000 sec, (b) a close view of final responses of systems.

Fig 11 shows a comparison of responses of RSAC and conventional MRAC in this test scenario. The results in the figure indicate that RSAC can provide an improved reference model following performance. This improvement mainly originates from the control optimizer block that synthesizes optimized control signals to strictly decrease model error. Conventional MRAC structure with MIT rule [8, 20] generates proportional control signal by using control error, and sharply changing of control error, which emerges at rising and falling edges of step reference signal, leads to high overshoots until MIT update rule responds for reduction of these overshoots. Delays in the response of MIT update rule causes the increase of the overshoot levels. In RSAC structure, sharp change in reference input cannot reduce control performance because reference input is not introduced to control optimizer. This is an important advantage of RSAC structure in practice.

## Experimental study

### Coaxial rotors experimental test platform

**Fig 12:**
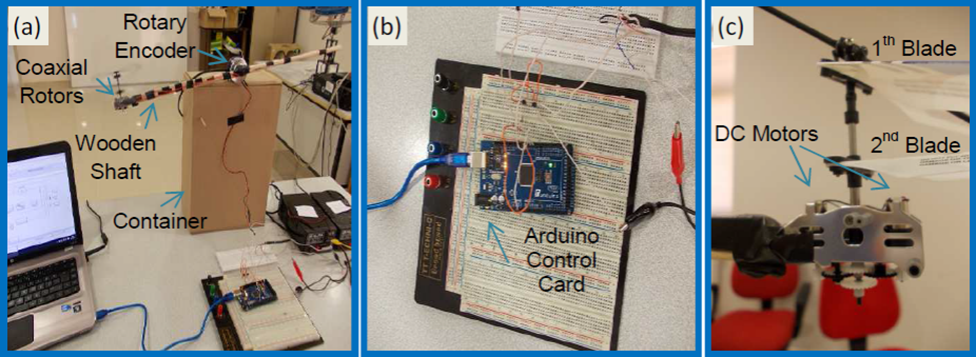
(a) A prototype of coaxial rotors control experimental test platform; (b) Close views of Arduino Mega 2560 card; (c) Close views of coaxial rotors and blades used in the experimental system.

Fig 12 depicts a prototype of rotors experimental test platform and its main components. Mechanical parts of this experimental test platform consist of a coaxial rotor, a wooden shaft, an incremental rotary encoder (ES5-0CCN 6942) and a container box.

**Fig 13:**
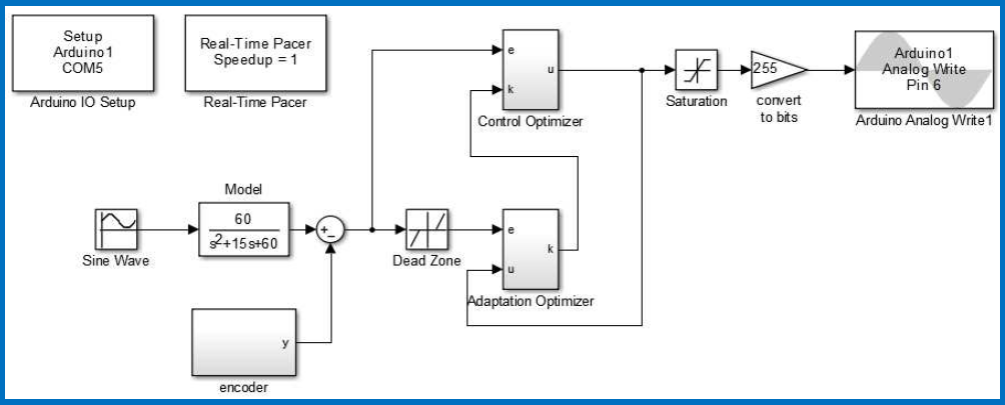
Matlab/Simulink design of the proposed adaptive control system.

**Fig 14:**
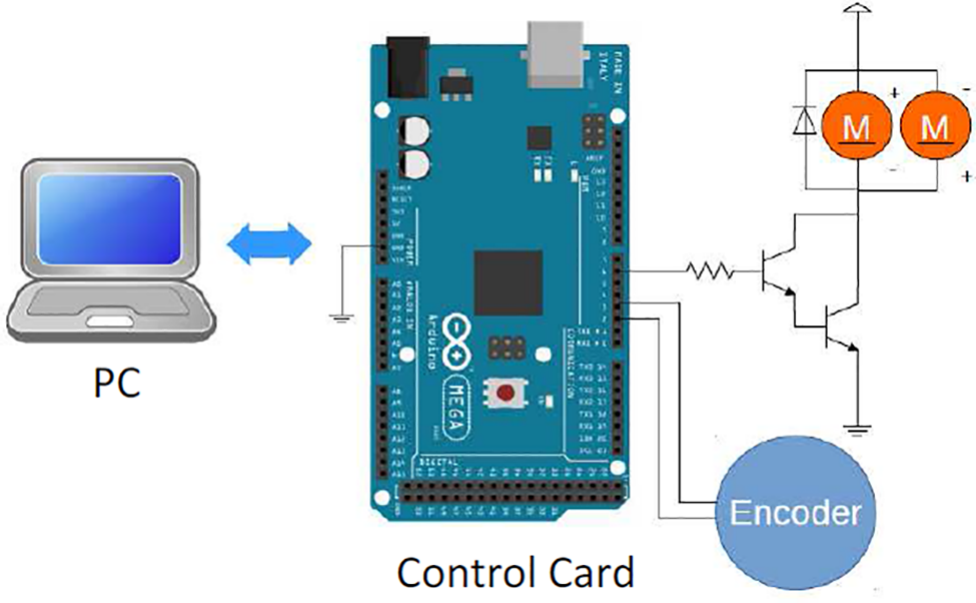
Electrical components of experimental system.

**Fig 15:**
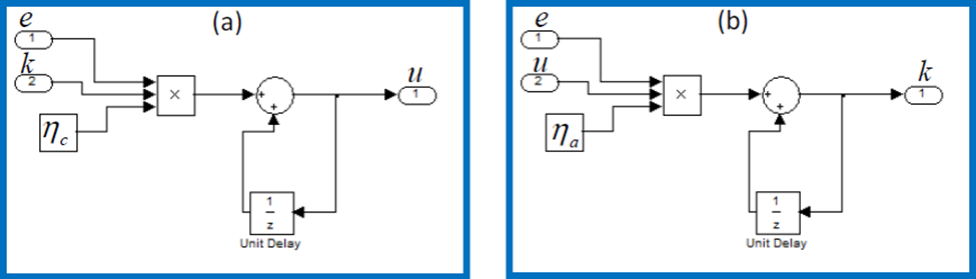
Implementation of update rules of (a) control optimizer (Eq 7) and (b) adaptation optimizer (Eq 11) in Simulink.

In this experimental study, we used standard coaxial rotors and blade set of LS-222 Gyro 3.5 Channel model helicopter. For online adaptive control of coaxial rotors test platform, Arduino Mega 2560 control card was used for data acquisition. The data captured from the encoder output and control signal applied to rotors by the Arduino control card. Arduino platforms are low-cost control card solutions and suitable for the low-cost experimental digital control studies. We have designed the RSAC structure in Matlab/Simulink environment as shown in Fig 13. Arduino control card receives the angle data of shaft from the rotary encoder and it drives two electric motors of coaxial rotors by means of a Darlington power amplification circuit. Fig 14 depicts the circuit diagram of electronic parts of the experimental system. Implementation of the update rules of the control optimizer (Eq 7) and the adaptation optimizer (Eq 11) in Simulink are illustrated in Fig 15.

### Experimental results

**Fig 16:**
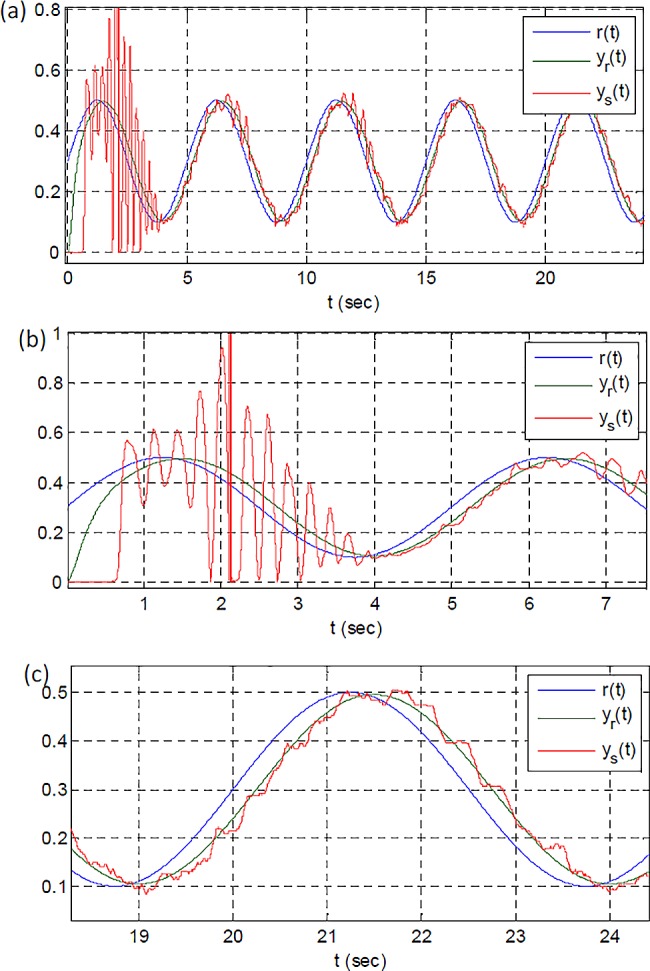
Sinusoidal reference input, output of reference model and output of coaxial rotors control; (a) a full view of experimental results; (b) a close view for initial response (Before adaptation); (c) a close view of final responses of the experimental system (After adaptation).

In the experimental study, the reference system model was taken as $T_{R}(s)=\frac{60}{s^{2}+15s+60}$. The error threshold was configured to *e*_*z*_ = 0.009. The initial value for learning rates and adaptation gain were set to (*η*_*a*_ = 0.01, *η*_*c*_ = 0.01) and *k*(0) = 0.05, respectively. A sinusoidal reference signal, *r*(*t*) = 0.3 + 0.2sin(0.1257*t*), is applied for the control of coaxial rotors. Fig 16 shows response of RSAC system. At the first period, shown in Fig 16(B), the output of control system cannot follow the output of reference system because adaptation to the controlled system has not occurred, yet. After the adaptation, improvement in the response of control performance manifests itself as more proper following the reference model output by control system as shown in Fig 16(C).

**Fig 17:**
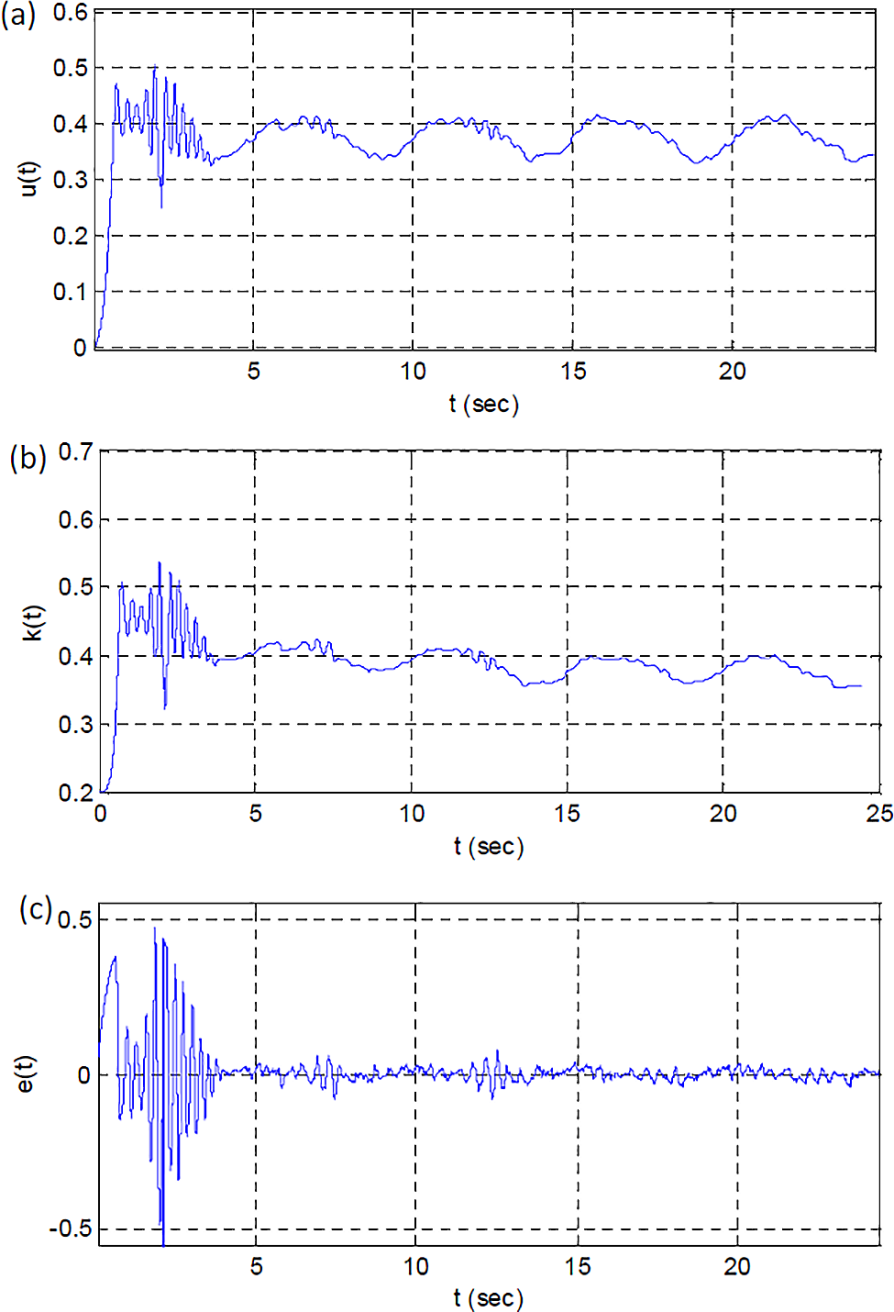
(a) The control signal, (b) the adaptation gain and (c) the model error signal from the experimental system.

Fig 17 shows change of control signal, adaptation gain and model error signal. The control signal and adaptation gain settle in the range of (0.3,0.4) and this is an indicator for completion of the adaptation process. The error signal also confirms a successful adaptation by decreasing the magnitude of ripples about the zero.

It should be noticed that the proposed method is sensitive to large dead-time system delay. The large system delays can lead to growing of output ripples. We observed that large dead time delays cause misleading of the update rules because of the update rules based on gradient descent require very recent values of error signals in order to correctly estimate gradient directions. The negative effects of large dead-time system delay on control performance should be considered in applications.

## Conclusions

In summary, this study introduces a RSAC structure, which employs gradient descent optimization technique for model reference adaptive control. Theoretical foundations and practical utilization of the RSAC structure were presented in the paper.

Some important remarks can be summarized as follows:

1. The proposed RSAC structure implements two numerical gradient descent optimization processes that work in conjunction. These processes are the control optimizer for control signal generation and the adaptation optimizer for estimation of instantaneous input-output relation of plant.
2. The design and implementation of the RSAC structure are quite straightforward. Update rules are fixed and simple equations. The proposed method does not require a proper modeling of plant dynamics and it is a noteworthy asset of the proposed method for control practice. Its computational scheme uses only an estimation of current value of time-varying adaptation gain that can characterize instantaneous input-output relation of a controlled system. The conventional MRAC mainly need mathematical modeling of plant to obtain effective update rules to tune coefficients of controller functions. For this reason, complexity of update rules of conventional MRACs depends on the plant function and it can reduce applicability of conventional MRAC structures.
3. Noise sensitivity of adaptation parameters and dynamic model mismatches are the major problems, that may decrease performance of adaptive control systems in real world applications. To deal with this problem, dead-zone modification for model error signal is a common approach.

In practice, very large system delay and high-amplitude noise signal may fail the adaptation process because large dead-time delay of error signal or high-amplitude noise on model error can easily mislead update rules. These effects cause to grow the amplitude ripples around set points and cause instability of the system in time. The future works should address solutions to decrease delay and noise sensitivity of RSAC.
